# Comparative analysis of the effects of opioids in angiogenesis

**DOI:** 10.1186/s12871-021-01475-7

**Published:** 2021-10-26

**Authors:** Tao Feng, Si Zeng, Jie Ding, Gong Chen, Bin Wang, Daguo Wang, Xueli Li, Kunfeng Wang

**Affiliations:** 1 Department of Anesthesiology, Affiliated Baoan Central Hospital of Guangdong Medical University, No 60 Leyuan Road, Baoan Distric of Shenzhen, Shenzhen, Guangdong Province China; 2 Department of Anesthesiology, Sichuan Academy of Medical Science & Sichuan Provincial People’s Hospital, Electronic Science and Technology University, 18 Huanhua Road, Chengdu, China

**Keywords:** Opioids, MAPK, μ-Opioid receptor, Angiogenesis

## Abstract

**Background:**

Angiogenesis, the formation of blood vessel from pre-existing ones, plays an important role in many pathophysiological diseases, such as cancer. Opioids are often used in clinic for the management of chronic pain in cancer patients at terminal phases. Here, we investigated and compared the effects and mechanisms of four opioids on angiogenesis.

**Methods:**

We performed angiogenesis assays on human umbilical vein endothelial cells (HUVEC) that represent an in vitro model to assess the toxicity of drugs to endothelium.

**Results:**

Morphine and oxycodone at 0.1 μM to 100 μM dose-dependently increased endothelial cell tube formation and proliferation. We observed the same in endothelial cells exposed to fentanyl at 0.1 μM to 10 μM but there was a gradual loss of stimulation by fentanyl at 100 μM and 1000 μM. Morphine and fentanyl reduced endothelial cell apoptosis-induced by serum withdrawal whereas oxycodone did not display anti-apoptotic effect, via decreasing Bax level. Oxycodone at the same concentrations was less potent than morphine and fentanyl. Different from other three opioids, codeine at all tested concentrations did not affect endothelial cell tube formation, proliferation and survival. Mechanism studies demonstrated that opioids acted on endothelial cells via μ-opioid receptor-independent pathway. Although we observed the increased phosphorylation of mitogen-activated protein kinase (MAPK) in cells exposed to morphine, fentanyl and oxycodone, the rescue studies demonstrated that the stimulatory effects of morphine but not fentanyl nor oxycodone were reversed by a specific MAPK inhibitor.

**Conclusion:**

Our work demonstrates the differential effects and mechanisms of opioids on angiogenesis.

**Supplementary Information:**

The online version contains supplementary material available at 10.1186/s12871-021-01475-7.

## Background

Angiogenesis, the formation of blood vessel from already established blood vessels, is required for tumor progression and metastasis [1]. Vascular endothelial growth factor (VEGF)-A and its receptor VEGFR-2, play an essential role in angiogenesis via various mechanisms, and inhibiting VEGF-VEGFR is a promising therapeutic approach for cancer [2]. Opioids are widely used medication for pain management in several medical conditions including cancer. Opioids act on central nervous system via binding and activating opioid receptors that express pain transmission and modulate pathways [3]. Opioid receptors are members of the G protein coupled receptor superfamily and are classified as μ, δ and κ. Apart from neuron cells, opioid receptors are found to be expressed in other types of cells, such as endothelial and immune cells, resulting in various systematic impacts [4, 5]. The effects of opioids on both cancer and angiogenesis are highly contentious as both pro- and anti- effects of tumor growth and neovascularization are reported [6, 7].

Morphine, fentanyl, oxycodone and codeine are μ-opioid receptor agonists but can bind to and activate δ and κ with differential affinity [8]. Among these four commonly used opioids, morphine has been mostly studied under preclinical settings for its direct effect on tumor cell and angiogenesis but the conclusions are contradictory [9–12]. Morphine stimulates angiogenesis under serum deprivation and oxidative stress conditions [9] whereas also suppresses angiogenesis associated with tumor growth in mice [13]. One study demonstrates that fentanyl stimulates angiogenesis in diabetic rats and promotes wound healing [14]. Another recent study using cell models reveals that fentanyl stimulates tumor angiogenesis [15]. The effects of oxycodone and codeine in angiogenesis are unknown. In this study, we systematically evaluated and compared the effects of all four opioids on tube formation, proliferation, migration and survival of endothelial cells. We further annotated the underlying mechanisms of opioids in endothelial cells.

## Methods

### Endothelial cell isolation and culture

Human umbilical vein endothelial cells (HUVECs) were isolated from human umbilical cord vein using the protocol described in Crampton et al’s work [16] with modification. Briefly, the fresh umbilical cords were collected from consented maternal ward patients at the Baoan Central Hospital of Shenzhen, which was approved by the institutional ethics approval committee. Adapters were inserted into vein at each end of the cord and secured tightly with silk thread. The vein was flushed with PBS and then incubated with 0.2% collagenase (Sigma) for 15 mins at room temperature. Collagenase solutions were warmed up to 37 °C in water bath prior to injecting into umbilical vein lumen. The released HUVECs together with collagenase were collected and spin down. The cell pelleted were resuspended with PBS and washed twice. Isolated HUVECs were cultured using CSC complete medium (Cell Systems) supplemented with 50 μg/ml gentamycin (Sigma) on 0.2% gelatine (Sigma)-coated flask. Cells were starved in basal CSC medium (Cell Systems) containing 2% FBS (starving medium) prior to all experiments.

### Drugs, antibodies and reagents

Morphine-HCL (Sintetica), fentanyl (Yichang Humanwell Pharmaceutical Co., Ltd) and oxycodone hydrochloride tablet (Sankyo Pharmaceutical Co. Ltd.) were obtained from the Department of Pharmacy, Baoan Central Hospital of Shenzhen. Codeine and PD98059 were obtained from Sigma and Selleckchem. Recombinant human VEGF was obtained from R&D Systems. p-Erk1/2(T202/T204), Erk1/2, Bax and Bcl-2 were purchased from Cell Signaling.

### In vitro angiogenesis assay

In vitro angiogenesis was performed using standard protocol [17]. Briefly, corning Matrigel growth factor reduced basement membrane matrix was thawed on ice. 100 ul was plated onto 96-well-plate and incubated at 37^0^ C for 1 h to allow the matrix solution to solidify. HUVECs at 2000 cells together with VEGF or drugs at different concentrations were suspended in starving medium and plated onto solidified matrix. After 6 h incubation in a tissue-culture incubator, capillary network structures were documented using an inverted microscope. Tube-like structure length was quantified by measuring the length of branches using Image J software.

### Proliferation assay

Cell proliferation was evaluated by bromodeoxyuridine (BrdU) incorporation method [18]. 10^4 cells were seeded into 96-well plate and incubated overnight. The next day, cells were starved for 3 h and drugs or VEGF were then added to the starving medium. After 24 h drug treatment, BrdU reagent was added into the culture media and proliferation was quantified using BrdU cell proliferation kit (Chemicon) according to manufacturer’s instructions.

### Boyden chamber migration assay

We performed migration assay using the Boyden chamber (Cell Biolabs) using the same protocol as described in our previous study [19]. Briefly, cells were placed onto upper chamber and drugs were added to lower chamber. After 8 h incubation in a tissue-culture incubator, the migrated cells were counted under microscope.

### Apoptosis assay

10^5 cells were seeded into 12-well plate and incubated overnight. The next day, cells were starved for 3 h and drugs or VEGF were then added to the starving medium. After 24 h drug treatment, apoptosis was determined by measuring cytosolic oligonucleosome-bound DNA [20] using a Cell Death ELISA kit (Roche) according to manufacture’s instructions.

### Western blot

10^6 cells were seeded into 6-well plate and incubated overnight. The next day, cells were starved for 3 h and drugs were then added to the starving medium. After 24 h drug treatment, cells were harvested for protein extraction using RIPA buffer. Western blot was performed using standard protocol [21]. Briefly, equal amount of proteins was loaded on SDS-polyacrylamide gel, resolved by electrophoresis, transferred to Hybond-C extra nitrocellulose membrane (Amershan-Pharmacia), probed with designated primary and secondary antibodies, and detected using SuperSignal substrate solution (Pierce).

### Statistical analyses

Statistical analyses were performed using unpaired Student’s t-test, with *P*-value < 0.05 as statistically significant. Comparison of three or more categorical variables were performed using ANOVA. All statistical analysis was done on the PRISM statistical software.

## Results

### Morphine, fentanyl and oxycodone but not codeine stimulate HUVEC capillary network formation

**Fig. 1:**
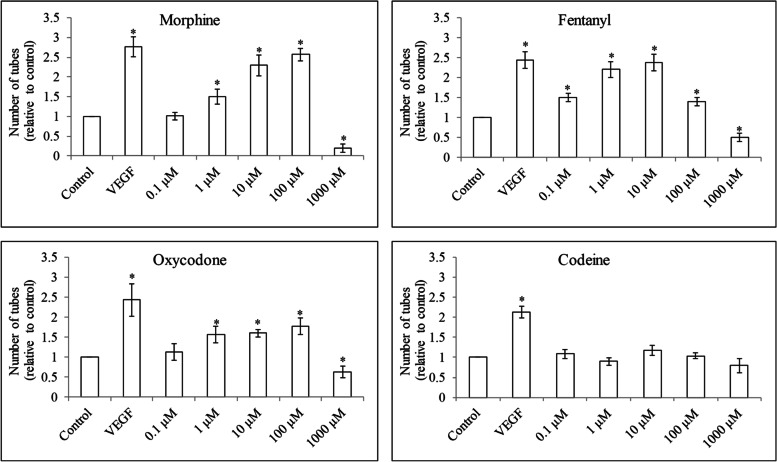
The effects of opioids in in vitro angiogenesis. Morphine, fentanyl and oxycodone at 0.1 μM to 100 μM increased capillary network formation whereas at 1000 μM decreased capillary network formation. Codeine at 0.1 μM to 1000 μM did not affect capillary network formation. VEGF at 15 ng/ml was used. Results shown are relative to control. Each experiment was repeated three times in triplicate, and each value indicates mean ± SD. *p < 0.05, compared to control

We evaluated the effects of four commonly used opioids on angiogenesis using in vitro Matrigel matrix angiogenesis assay. HUVECs represent an in vitro model to assess the toxicity of drugs to endothelium and are relevant to the process of pathophysiological angiogenesis [22]. We plated HUVECs on the growth factor reduced basement membrane matrix and used this as control. As a comparison, we included a sample with the addition of 15 ng/ml VEGF as positive control. Consistent with the previous report [23], we demonstrated that VEGF increased HUVEC tube formation by ~ 2.5 fold compared with control (Fig. 1 and Figure S1 to S4). The addition of morphine at concentrations ranging from 0.1 μM to 100 μM increased HUVEC tube formation. Oxycodone at the same concentrations significantly increased HUVEC tube formation. Fentanyl at 0.1 μM, 1 μM and 10 μM increased HUVEC tube formation. Morphine, fentanyl and oxycodone at 10 μM stimulates tube formation by ~ 2.3, ~ 2.3 and ~ 1.6 fold, respectively, suggesting that morphine and fentanyl are more potent than oxycodone. Interestingly, morphine and oxycodone at 1000 μM sharply decreased tube formation. There was a gradual loss of stimulation of tube formation by fentanyl at 100 μM and 1000 μM. In contrast, codeine at all tested concentrations did not affect tube formation at all. Our results demonstrate that three out of four tested opioids display pro-angiogenic activities.

### Four opioids display differential effects on HUVEC growth, migration and survival

**Fig. 2:**
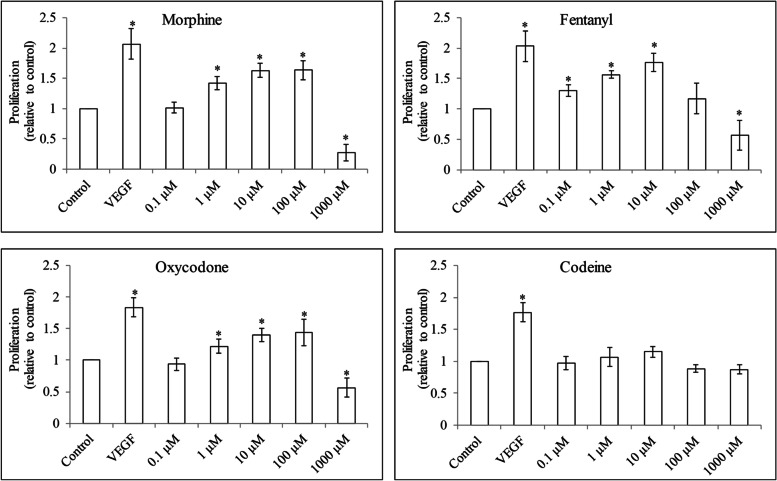
The effects of opioids in HUVEC proliferation. Morphine, fentanyl and oxycodone at 0.1 μM to 100 μM increased proliferation whereas at 1000 μM decreased proliferation in HUVECs. Codeine at 0.1 μM to 1000 μM did not affect HUVEC proliferation. VEGF at 15 ng/ml was used. Results shown are relative to control. Each experiment was repeated three times in triplicate, and each value indicates mean ± SD. *p < 0.05, compared to control

To evaluate if opioids stimulate HUVEC proliferation, we assessed BrdU level after 24 h opioid treatment under serum-reduced culture condition. Consistent with the stimulated angiogenesis, VEGF increased HUVEC proliferation by 2-fold compared with control (Fig. 2). We observed that morphine and oxycodone at 0.1 μM to 100 μM increased cell proliferation by 1.6- and 1.4-fold, respectively. Fentanyl at 0.1 μM to 10 μM increased proliferation by 1.8-fold but at 100 μM and 1000 μM demonstrated gradual loss of proliferation stimulation. Morphine, fentanyl and oxycodone at 1000 μM decreased cell proliferation. Codeine at all tested concentrations did not affect HUVEC proliferation.

**Fig. 3:**
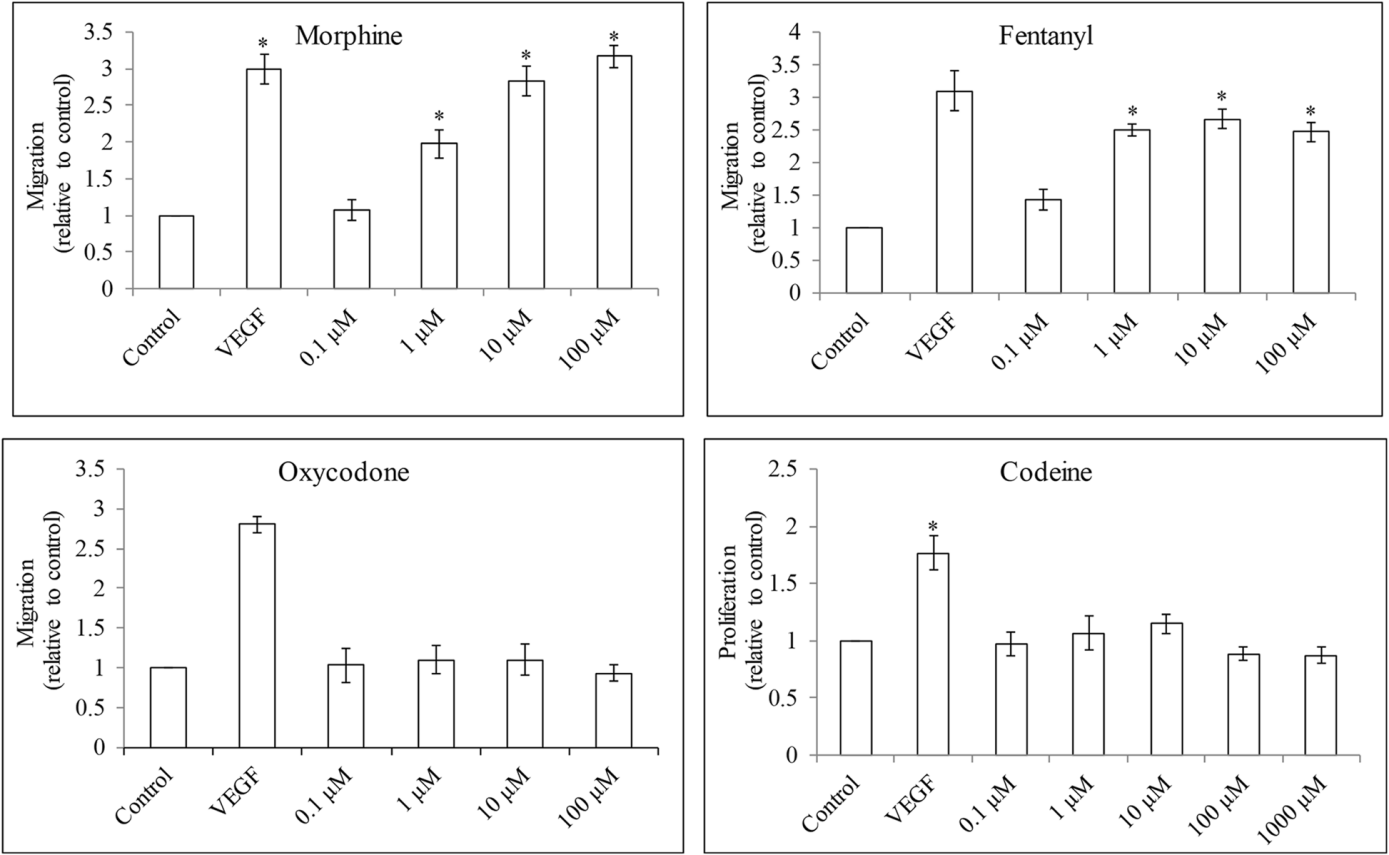
The effects of opioids in HUVEC migration. Morphine, fentanyl and oxycodone at 0.1 μM to 100 μM increased migration in HUVECs. Codeine at 0.1 μM to 100 μM did not affect HUVEC migration. VEGF at 15 ng/ml was used. Results shown are relative to control. Each experiment was repeated three times in triplicate, and each value indicates mean ± SD. *p < 0.05, compared to control

**Fig. 4:**
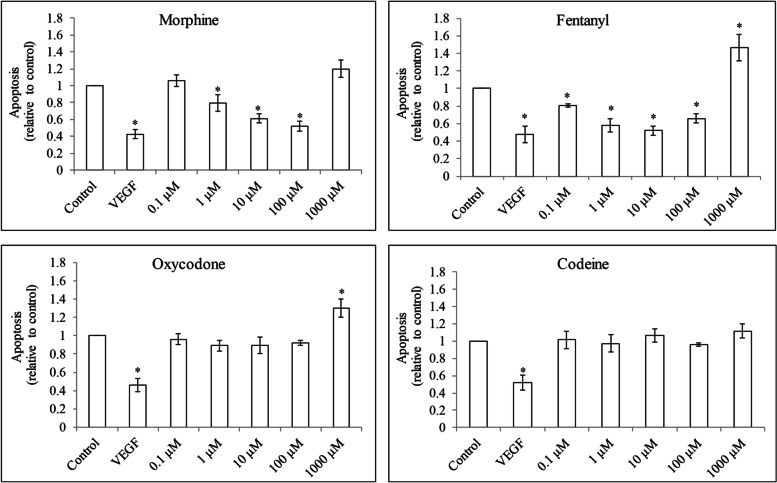
The effects of opioids in HUVEC apoptosis. Morphine, fentanyl and oxycodone but not codeine at 0.1 μM to 1000 μM decreased apoptosis. VEGF at 15 ng/ml was used. Results shown are relative to control. Each experiment was repeated three times in triplicate, and each value indicates mean ± SD. *p < 0.05, compared to control

**Fig. 5:**
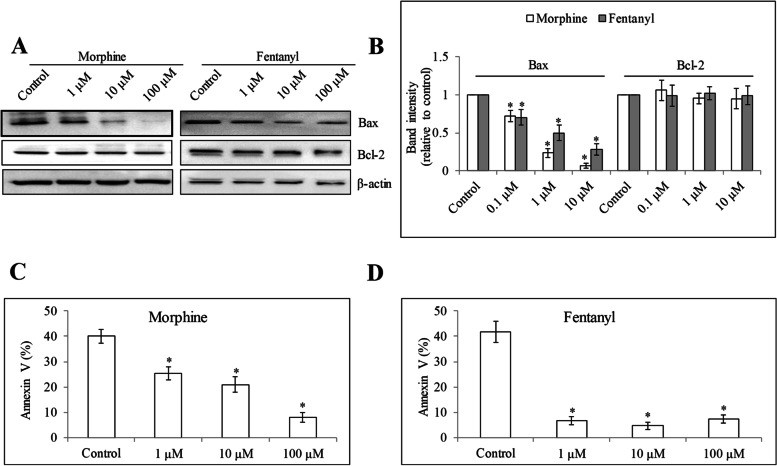
The effects of opioids in HUVEC apoptotic proteins. Western blot (**A**) and quantification of band intensity (**B**) of Bax and Bcl-2 in HUVEC after 24 h treatment of morphine and fentanyl. Morphine (**C**) and fentanyl (**D**) at 1, 10 and 100 μM significantly decreases Annexin V percentage in HUVEC. Each experiment was repeated three times in triplicate, and each value indicates mean ± SD. *p < 0.05, compared to control

To evaluate the effect of opioids on HUVEC migration, we performed three-dimensional Boyden chamber migration assay which is informative and similar to an in-vivo migration context. We found that morphine and fentanyl but not oxycodone or codeine at 0.1 μM to 100 μM significantly increased HUVEC migration (Fig. 3 and Figure S5). We further evaluated if opioids can protect HUVEC from apoptosis induced by growth factor reduction, we assessed oligonucleosomal DNA fragments after 24 h opioid treatment under growth factor-reduced culture condition. As a positive control, VEGF decreased HUVEC apoptosis compared with control (Fig. 4). Morphine and fentanyl but not oxycodone at 0.1 μM to 100 μM decreased apoptosis. In contrast, morphine, fentanyl and oxycodone at 1000 μM increased HUVEC apoptosis. Codeine at all tested concentrations did not affect HUVEC apoptosis. Morphine and fentanyl at 1, 10 and 100 μM significantly decreased pro-apoptotic protein Bax without affecting Bcl-2 level (Fig. 5A and B). Consistently, Annexin V staining was significantly decreased in cells exposed to morphine and fentanyl (Fig. 5C and D and Figure S7). These clearly indicate the anti-apoptotic effect of morphine and fentanyl. Taken together, our results demonstrate the differential effects of four opioids on HUVEC growth, migration and survival.

### Opioids act on HUVEC in a μ-opioid receptor-independent manner

**Fig. 6:**
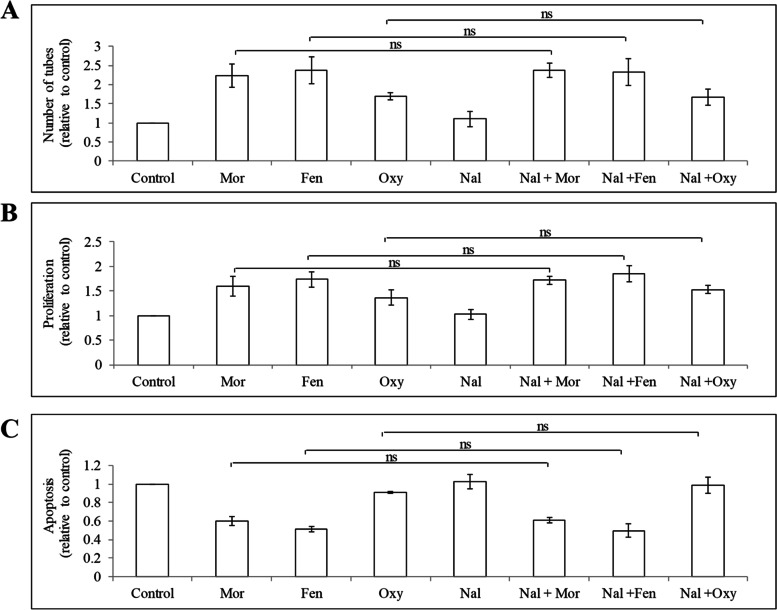
The effects of opioids in HUVEC are opioid receptor-independent. Naloxone did not affect the effects of morphine, fentanyl and oxycodone in HUVEC capillary network formation (**A**), proliferation (**B**) and apoptosis (**C**). Naloxone at 10 μM was used. Mor, morphine; Fen, fentanyl; Oxy, oxycodone; Nal, naloxone. Results shown are relative to control. Each experiment was repeated three times in triplicate, and each value indicates mean ± SD

Morphine, fentanyl and oxycodone are known μ-opioid receptor agonists. To investigate whether μ-opioid receptor is involved in these opioids’ action in endothelial cells, we treated HUVEC with opioids in the absence or presence of naloxone which is a a μ-opioid receptor antagonist) [24] and examined cell tube formation, proliferation and apoptosis. The concentration of naloxone used in our work is referred from the established reported literatures [25, 26]. We found that naloxone did not antagonize morphine, fentanyl or oxycodone-induced capillary network formation (Fig. 6A) or endothelial cell growth (Fig. 6B). Naloxone did not antagonize morphine and fentanyl’s anti-apoptosis effects in HUVEC (Fig. 6C). These demonstrate that opioids act on endothelial cells in a μ-opioid receptor-independent manner. This is also consistent with our observation that naloxone alone did not affect endothelial cell tube formation, growth or survival (Fig. 4).

### Morphine but not fentanyl or oxycodone stimulates angiogenesis via MAPK activation

**Fig. 7:**
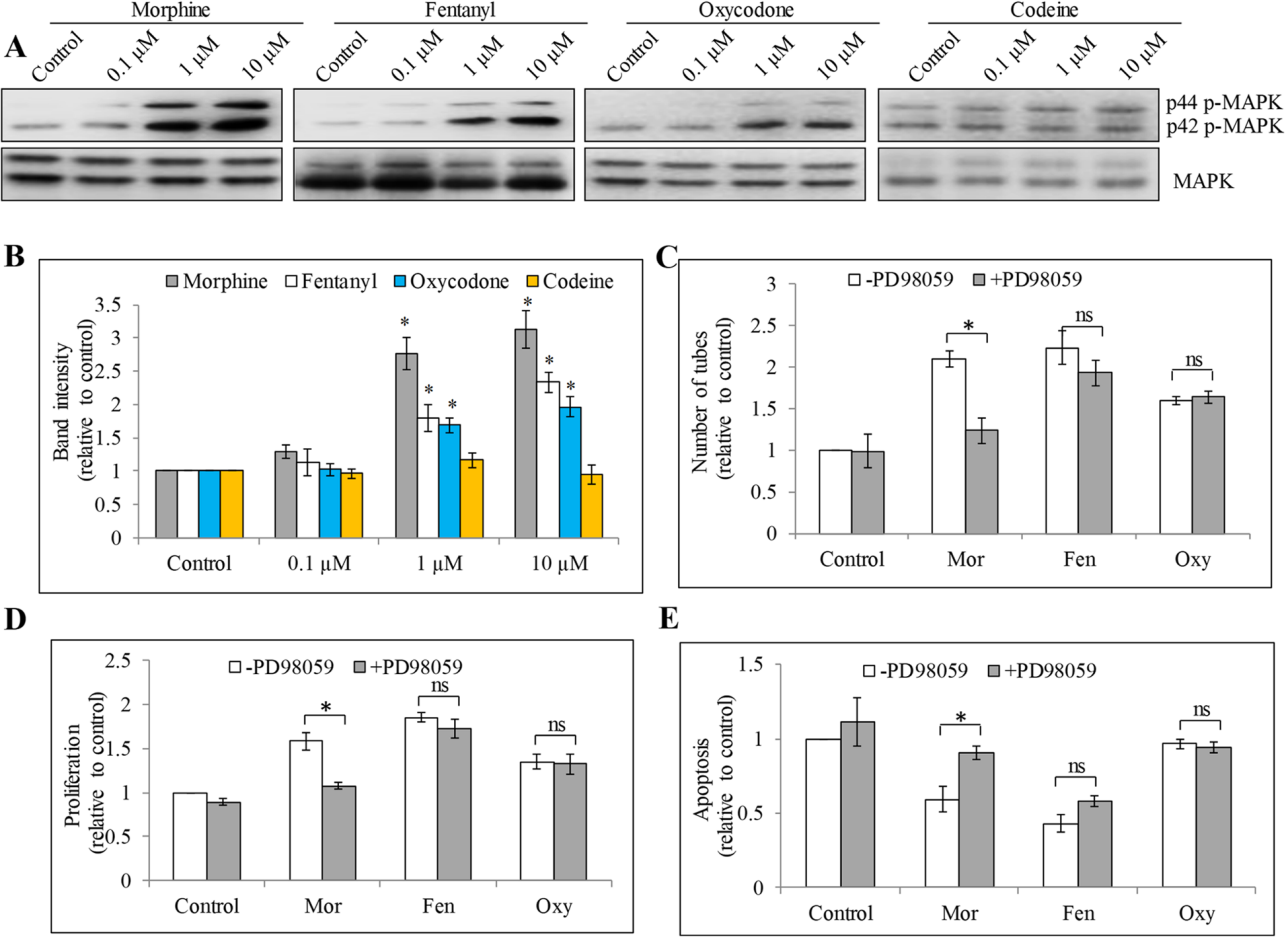
Morphine but not fentanyl or oxycodone acts on HUVEC via MAPK activation. Western blots (**A**) and quantification of band intensity (**B**) of HUVEC exposed to morphine, fentanyl, oxycodone or codeine for 6 h. MAPK inhibitor PD98059 (1 μM) significantly reversed the effects of morphine but not fentanyl or oxycodone in stimulating capillary network formation (**B**), increasing proliferation (**C**) and decreasing apoptosis (**D**) in HUVEC. Results shown are relative to control. Each experiment was repeated three times in triplicate, and each value indicates mean ± SD. *p < 0.05, compared to -PD98059. ns, not significant. Relevant blot images were cropped to improve clarity

Morphine and opioid receptor agonists have been shown to induce MAPK activation in endothelial cells to promote proliferation [27, 28]. Given our findings above, we performed western blot analysis of phosphor- and total p44/42 MAPK in HUVEC exposed to opioids. We found that morphine, fentanyl and oxycodone at 0.1 μM, 1 μM and 10 μM increased p-p44/42 MAPK without affecting level of total MAPK (Fig. 7A, B and Figure S8). Codeine did not affect phosphor- nor total p44/42 MAPK (Fig. 7A, B and Figure S8). We performed rescue experiments using specific MAPK inhibitor PD98059 at concentration that has been shown to effectively block MARK signaling [28]. We found that PD98059 blocked morphine-induced endothelial cell tube formation, growth and survival (Fig. 7C to E). Interestingly, PD98059 did not rescue the pro-angiogenic, pro-proliferative and anti-apoptotic effects of fentanyl and oxycodone on endothelial cells (Fig. 7C to E). These results demonstrate that morphine but not fentanyl or oxycodone acts on endothelial cells via MAPK activation. We also performed immunoblotting analysis of angiogenic markers (eg, CD31, E-selection) and growth factor FGF in endothelial cells after opioids treatment. We found that all tested opioids did not affect CD31, E-selection and FGF levels in HUVEC (Figure S9 and S10).

## Discussion

Although substantial evidence have highlighted that perioperative anesthetic management of cancer patients could potentially affect longer-term recurrence and metastases [29], this requires confirmation in prospective, randomized clinical trials which will take many years to obtain data. Most studies have been conducting preclinical research using cell culture and animal models to investigate the direct role of anesthetic agents on cancer. In line with these efforts, we previously reported that sevoflurane, a volatile anesthetic agent, displays anti-cancer activities in cervical cancer [19]. Opioids are most commonly used medication for perioperative pain as well as cancer pain in cancer patients [30]. As cancer progression is largely dependent on angiogenesis, it is important to understand the influence of opioids on angiogenesis in order to guide the proper clinical use of opioids in cancer patients, particularly given that conclusions on the effects of opioids are contradictory. In this work, we show that three out of four opioids have stimulatory effects on angiogenesis, and furthermore that opioids stimulate angiogenesis via different mechanisms.

We firstly demonstrated that morphine at 0.1 μM to 100 μM stimulated endothelial cell tube formation, proliferation and survival under growth factor-reduced condition. Among the published research studies regarding the effects of morphine on neovascularization, stimulatory effect was observed in one half [11, 31] and inhibitory effect was observed in another half [13, 32]. The reason behind this discrepancy is unclear, but it may be a result of differences in model systems, endothelial type, drug concentrations and experimental conditions. In support of this possibility, our finding is consistent with the recent study by Zhang et al. that morphine stimulates angiogenesis after serum deprivation [9] because our experimental settings are similar to Zhang et al’s work. We next showed that oxycodone at the same concentration range also displayed pro-angiogenic activity, but was less potent than morphine. Fentanyl at 0.1 μM to 10 μM stimulates angiogenesis, which is supported by the recent study [15]. In addition, our findings further extend the previous study that fentanyl at higher concentrations (eg, 100 μM and 1000 μM) gradually lost its stimulatory activity. The sharply reduced angiogenesis was observed in morphine, fentanyl and oxycodone at 1000 μM, suggesting that opioids at this concentration is likely toxic to endothelial cells. Codeine behaves differently on endothelial cells. We show that codeine up to 1000 μM does not affect endothelial cell biology activities at all. In contrast, morphine and fentanyl demonstrate potent pro-angiogenic activities, and oxycodone demonstrates moderate pro-angiogenic activities. Using in vitro angiogenesis models, we and others consistently demonstrate the pro-angiogenetic effects of morphine and fentanyl [15, 28]. It is worthy of confirming the effects of opioids on angiogenesis in vivo.

Although published studies have shown that the pro-angiogenic and anti-angiogenic activities of opioids were opioid-receptor based [7], our finding demonstrates that morphine acts on endothelial cells via μ-opioid receptor-independent MAPK activation which is consistent with the previous report [28]. In contrast, fentanyl and oxycodone act on endothelial cells via other μ-opioid receptor-independent mechanisms. Our findings that naloxone neither antagonizes opioids-induced angiogenesis nor induces angiogenesis by itself are also observed in human microvascular endothelial cells exposed to morphine and naloxone [28]. We further demonstrate that morphine, fentanyl and oxycodone but not codeine increase phosphorylation of p44/42 MAPK. A specific MAPK inhibitor reverses the effects of morphine but not fentanyl or oxycodone, indicating that only morphine acts on endothelial cells via MAPK activation and furthermore that fentanyl and oxycodone are via other mechanisms. Studies suggest that MAPK pathway is one of the downstream events of opioid receptor activation in endothelial cells [7]. Our work demonstrates that morphine activates MAPK in an opioid receptor-independent manner. MAPK is activated by receptor tyrosine kinases, G protein–coupled receptors and ion channels [33], which might be the upstream target of morphine. A recent work reveals that fentanyl simulates angiogenesis via promoting VEGFR2/FAK/PI3K/Akt pathway and increasing activities of small GTPases [15]. We speculate that VEGFR2-mediated signaling might be involved in fentanyl’s action. Oxycodone has been shown to stimulate cancer cell biological activities via regulating EGFR pathway [34]. Given the importance of EGFR in endothelial cells, we speculate that oxycodone might increase angiogenesis via promoting EGFR-mediated signaling.

We note that although morphine, fentanyl and oxycodone stimulate angiogenesis, the mechanisms of their action are different: morphine acts on endothelial cells via μ-opioid receptor-independent MAPK activation whereas fentanyl and oxycodone act on endothelial cells via other μ-opioid receptor-independent mechanisms. Although morphine, fentanyl, oxycodone and codeine belong to opioid family, they are independent drugs in aspects of their effects and underlying mechanisms on angiogenesis. The reasons on why codeine does not affect endothelial cell biology whereas the rest of the other three do are not clear. We speculate that the difference is less likely due to their activity on opioid receptor and is more likely due to their unrecognized pleiotropic effects.

In conclusion, our work on the comparative analysis of four opioids in endothelial cells demonstrates that opioids have differential effects on angiogenesis and there is no common mechanism for their action. Our work is the first to reveal the stimulatory effect of oxycodone on angiogenesis. The elaboration of mechanism action of each opioid in angiogenesis is worthy of further investigation.

## Supplementary Information

**Additional file 1.**

## Data Availability

The datasets used and/or analysed during the current study available from the corresponding author on reasonable request.

## Abbreviations

HUVEC: Human umbilical vein endothelial cell
VEGF: Vascular endothelial growth factor
EGFR: Epithelial growth factor receptor
VEGFR: Vascular endothelial growth factor receptor
MAPK: Mitogen-activated protein kinase
BrdU: Bromodeoxyuridine.
