# Supplementary material for: Comparative analysis of the effects of opioids in angiogenesis
Source: BMC Anesthesiol. 2021 Oct 26;21:257. doi: 10.1186/s12871-021-01475-7 (PMC8549314; doi:10.1186/s12871-021-01475-7)
Supplement: Supplementary file 1 — Additional file 1. [file 12871_2021_1475_MOESM1_ESM.doc]

**Comparative analysis of the effects of opioids in angiogenesis**

**Fig. S1 Representative images of *in vitro* angiogenesis on HUVEC after morphine treatment.**

**Fig. S2 Representative images of *in vitro* angiogenesis on HUVEC after fentanyl treatment.**

**Fig. S3 Representative images of *in vitro* angiogenesis on HUVEC after oxycodone treatment.**

**Fig. S4 Representative images of *in vitro* angiogenesis on HUVEC after codeine treatment.**

**Fig. S5 Representative images of Boyden chamber migration assay on HUEVC after opioid treatment.** VEGF at 15ng/ml, morphine at 100 µM, fentanyl at 100 µM, oxycodone at 100 µM and codeine at 100 µM were used.

**Fig. S6 Original images of Western blot of Fig 5A.**

**Fig. S7 Representative FACS images of Annexin V/7-AAD staining on HUVEC after morphine and fentanyl treatment.**

**Fig. S8 Original images of Western blot of Fig 7A.**

**Fig. S9 Western blots of HVUEC exposed to morphine, fentanyl, oxycodone or codeine.**

**Fig. S10 Original images of Western blot of Fig. S9.**
